# Visualizing radiological data bias through persistence images

**DOI:** 10.18632/oncotarget.28670

**Published:** 2024-11-12

**Authors:** Yashbir Singh, Colleen Farrelly, Quincy A. Hathaway, Gunnar Carlsson

**Keywords:** persistence images, data interpretation, radiology, topology

## Abstract

Persistence images, derived from topological data analysis, emerge as a powerful tool for visualizing and mitigating biases in radiological data interpretation and AI model development. This technique transforms complex topological features into stable, interpretable representations, offering unique insights into medical imaging data structure. By providing intuitive visualizations, persistence images enable the identification of subtle structural differences and potential biases in data acquisition, interpretation, and AI model training. Persistence images can also facilitate stratified sampling, matching statistics, and noise filtration, enhancing the accuracy and equity of radiological analysis. Despite challenges in computational complexity and workflow integration, persistence images show promise in developing more accurate, equitable, and trustworthy AI systems in radiology, potentially improving patient outcomes and personalized healthcare delivery.

## INTRODUCTION

In the ever-evolving landscape of medical imaging and artificial intelligence (AI), the challenge of identifying and mitigating biases in radiological data interpretation remains paramount. As we strive for more accurate and equitable healthcare outcomes, an innovative approach from topological data analysis (TDA) has emerged: persistence images. This technique offers a promising avenue for visualizing complex radiological data and uncovering hidden biases that may influence diagnosis and treatment decisions.

### Understanding persistence images

Persistence images are a stable vector representation of topological features derived from persistence diagrams, which are core tools in TDA [1]. While persistence diagrams capture the birth and death of topological features across different scales, persistence images transform this information into a format more amenable to machine learning techniques [2]. The key advantage of persistence images lies in their stability: small perturbations in the input data result in only small changes in the persistence image [3]. This property makes them particularly valuable in medical imaging, where variations in image acquisition or patient positioning should not dramatically alter the underlying topological features being analyzed.

### Interpretable visualizations of complex radiological data

One of the most significant benefits of persistence images is their ability to provide interpretable visualizations of complex radiological data. By encoding topological features as intensity values in a 2D image, persistence images offer a more intuitive representation of the data’s structure compared to raw persistence diagrams or traditional statistical summaries [4]. In the context of radiology, these visualizations can help highlight subtle structural differences in images that might be indicative of specific pathologies or biases in data interpretation. For instance, persistence images could reveal consistent differences in the topological features of lung CT scans between different demographic groups, potentially uncovering biases in how these images are acquired [5].

### Identifying and addressing biases

The use of persistence images in radiological analysis opens up new possibilities for identifying and addressing biases in both data interpretation and AI model training:

#### Matching statistics and stratified sampling

Persistence images can be used to compute matching statistics between different subsets of radiological data. This approach allows for a more nuanced comparison of data distributions, helping to identify potential biases in sampling or data collection [6]. By visualizing these matching statistics through persistence images, researchers and clinicians can more easily identify underrepresented groups or oversampled categories in their datasets. This insight can then inform stratified sampling strategies to create more balanced and representative training sets for AI models, mitigating class bias and improving overall model performance; further, this can better inform clinicians and researchers about the limitations of a model’s generalizability [7].

#### Mitigating implicit bias in interpretation

Persistence images offer a powerful tool for breaking down radiological data by various demographic factors. By generating and comparing persistence images for different groups (e.g., age, gender, ethnicity), we can visually identify systematic differences in image features that might be indicative of bias in human interpretation [8]. For example, in patients with perceived low risk of breast cancer (e.g., young, no hormone replacement therapy, negative family history, no germline mutation) [9] , persistence images can offer an unbiased view of breast morphology and the likelihood of an asymmetry being malignant. Supporting clinicians in cases where implicit biases (such perceived low risk patients) may occur will ultimately make interpretation more equitable [10].

#### Filtration of noise

One of the inherent strengths of persistence images is their ability to filter out noise while preserving meaningful topological features. This property is particularly valuable in radiological imaging, where image artifacts and noise can significantly impact interpretation [11]. By focusing on persistent topological features and representing them in a stable format, persistence images can help radiologists and AI models distinguish between genuine anatomical structures and noise or artifacts. This filtration effect can lead to more reliable and less biased interpretations of complex imaging data [12].

### Applications in AI model training and evaluation

The vector representation provided by persistence images makes them particularly well-suited for integration into machine learning pipelines. This opens up several exciting possibilities for improving AI model training and evaluation in radiology:

1. Feature engineering: Persistence images can serve as topological feature vectors, complementing traditional image features in AI model training. This can lead to more robust models that are sensitive to both local and global structural characteristics of medical images [13].
2. Model interpretability: By visualizing the persistence images of the input data alongside model predictions, we can gain insights into which topological features are most influential in the model’s decision-making process. This enhanced interpretability can help identify potential biases in the model’s behavior [14].
3. Quality assurance: Persistence images can be used as a quality assurance tool for both input data and model outputs. By comparing the persistence images of model predictions with those of ground truth data, we can quickly identify systematic errors or biases in the model’s performance across different patient subgroups [15].

### Challenges and future directions

While persistence images offer significant promise in visualizing and addressing biases in radiological data, several challenges remain:

1. Computational complexity: Generating persistence images for large-scale radiological datasets can be computationally intensive. Developing more efficient algorithms and leveraging high-performance computing resources will be crucial for widespread adoption [16].
2. Integration with existing workflows: Incorporating persistence image analysis into established radiological workflows will require careful planning and validation. User-friendly tools and interfaces will be essential to make this technology accessible to clinicians who may not have expertise in TDA [17].
3. Standardization: As with any new analytical technique, establishing standards for generating, interpreting, and comparing persistence images across different institutions and imaging modalities will be crucial for ensuring reproducibility and comparability of results [18].

## CONCLUSIONS

Persistence images represent a powerful new tool in our ongoing efforts to visualize, understand, and mitigate biases in radiological data interpretation and AI model development. By providing stable, interpretable visualizations of complex topological features, they offer unique insights into the structure of medical imaging data that can complement existing analytical approaches. As we continue to refine and validate this technique, persistence images have the potential to play a crucial role in developing more accurate, equitable, and trustworthy AI systems in radiology. By helping us visualize and address hidden biases, they can contribute to improved patient outcomes and more personalized healthcare delivery. The journey towards truly unbiased radiological analysis is ongoing, but with innovative approaches like persistence images, we are illuminating the path forward, one topological feature at a time.
